# Hookups, hashtags, and health: A Latent Class Analysis of social media, mental health and sleep among MSM in Paris, France

**DOI:** 10.1016/j.ssmmh.2026.100615

**Published:** 2026-03-20

**Authors:** Cho-Hee Shrader, Su Hyun Park, Jenesis Merriman, Melissa DuPont-Reyes, Nour Makarem, Dustin T. Duncan

**Affiliations:** a Rutgers, The State University of New Jersey, School of Nursing, 180 University Ave, Rm 320, Newark, NJ, 07102, USA; b Saw Swee Hock School of Public Health, National University of Singapore and National University Health System, Singapore, 117549, Singapore; c Department of Sociomedical Sciences, Columbia University Mailman School of Public Health, 722 West 168th Street, New York, NY, 10032, United States of America; d Department of Epidemiology, Columbia University Mailman School of Public Health, 722 West 168th Street, New York, NY, 10032, United States of America

**Keywords:** Sexual and gender minorities, Social media, Sleep, Mental health, France

## Abstract

**Background:**

Social media use is ubiquitous, permeating nearly all populations globally, including men who have sex with men (MSM), despite there being mixed impacts to its use. To fill this gap, we sought to typologize social media usage, mental health, and sleep health among MSM in Paris, France, a city with a large LGBTQIA + community and high social media usage.

**Methods:**

Participants were recruited using broadcast advertisements on a popular geosocial networking application in Paris in October 2016. Consenting participants completed a self-administered survey online (French or English) that assessed: sleep characteristics; mental health; and social media use behaviors. We conducted a Latent Class Analysis (LCA) to characterize the number of distinct latent classes across the three primary constructs of interest.

**Results:**

A total of 580 participants completed the survey and 539 (92.9%) provided complete data for the LCA. The four-class model fit our data best; thus, we assigned participants to one of four distinct latent classes: 1) *MSM using social media for sex with stress and poor sleep* (n = 97), 2) *MSM using social media generally with good mental and sleep health* (n = 172), 3) *MSM using social media for sex with good mental and sleep health* (n = 147), and 4) *MSM using social media for sex with distress and poor sleep* (n = 123).

**Conclusion:**

Findings from this work may inform culturally-tailored interventions, public health messaging, and policy efforts to promote mental, behavioral, and digital health among MSM in France and beyond, while identifying opportunities to leverage digital platforms for sleep and mental health in digital communities.

## 1. Introduction

Social media use is now ubiquitous, permeating nearly all populations globally, including men who have sex with men (MSM) (Baral et al., 2018; Broaddus et al., 2015; Chiu and Young, 2015; Gao et al., 2023; Krishnan et al., 2018; Kudrati et al., 2021; Patel et al., 2016; Wang et al., 2020; Zheng et al., 2020) despite there being mixed impacts to its use (Kanchan and Gaidhane, 2023). In 2024, there were an estimated 5.04 billion social media users, composing approximately 62.3% of the global population (Kemp & Datareportal, 2025). More specifically in France, there were an estimated 50.4 million active social media users, composing 75.7% of the nation's population (Kemp, 2025). The European Men-Who-Have-Sex-With-Men Internet Survey (EMIS-2017) found that among 10,996 MSM recruited from France, the specific social media apps that the sample was recruited from included 44.1% from Romeo, 25.1% from Hornet, and 19.3% from Grindr (Weatherburn et al., 2020), suggesting that MSM engage with a wide array of social media platforms. These platforms can also include Twitter, Facebook, Instagram, TikTok (Hiebert and Kortes-Miller, 2021), and Snapchat, and are not only used for social connection and information sharing but also as tools to facilitate romantic and sexual encounters (“hookups”) (Broaddus et al., 2015; Chiu and Young, 2015; Gao et al., 2023; Goedel and Duncan, 2015, 2016; Kanchan and Gaidhane, 2023). Social media may be used for a number of other reasons, including boredom (which encourages engagement in social media via hashtags and commenting on photos and videos) making it appealing, in addition to negative health outcomes such as to find drugs and use drugs (Goedel and Duncan, 2016).

In 2025, the Evaluation of Digital Addiction study found that over 50% of French adults use social media for 1.5 h or more a day, 66.7% of French adults exhibited compulsive smartphone use, and 36.7% of adults were indicated for smartphone addiction (Vogel et al., 2021). Alarmingly, higher smartphone compulsions scores were associated with risky behaviors such as texting while driving and sleep disturbances (Vogel et al., 2021); one study among youth also suggests that sleep quality and duration may mediate the relationship between social media use and mental health (Bérard et al., 2023). Additionally, previous studies among MSM in Western Europe have identified relationships between financial hardship (Duncan et al., 2017a), neighborhood safety (Duncan et al., 2017b), stress (Mountcastle et al., 2018), and sexually explicit text messaging (“sexting”) (Al-Ajlouni et al., 2019) on the outcome of poor sleep quality. While the role of social media in shaping sexual networks, impacting mental health, and leveraging HIV-related risk behaviors among MSM has been well-documented in certain regions such as the United States (Broaddus et al., 2015; Chiu and Young, 2015; Filice et al., 2020; Goedel and Duncan, 2015; Kudrati et al., 2021) and China (Gao et al., 2023; Wang et al., 2020; Zheng et al., 2020), the broader health implications of social media use for this population remain underexplored for a wide range of health outcomes, including psychosocial and behavioral health outcomes such as anxiety, depression, or sleep disturbances (Ibrahim et al., 2024). This knowledge gap is especially pronounced in European urban centers like Paris, where digital platforms may intersect uniquely with cultural, structural, and policy environments influencing MSM's health and well-being.

Despite its pervasive influence, limited research on the relationship between social media use behaviors, mental health, and sleep health of MSM exists, particularly within Western Europe. To help deepen how our understanding of how social media usage, mental health, and sleep health may typologize, a Latent Class Analysis (LCA) may be useful. LCA can provide a nuanced characterization of how sets of responses among participants may typologize and form a pattern (Collins and Lanza, 2009; Jung and Wickrama, 2008; Weller et al., 2020; Wilson and Urick, 2022). For example, LCA has revealed that sleep quality can be described as “poor” or “good” with a Pittsburgh Sleep Quality Index greater than or equal to 5 indicating poor sleep quality (Yildirim and Boysan, 2017). Previous LCAs among MSM have focused on social norms and influences on sexual health (Shrader et al., 2023), sexual partner typologies (Janulis et al., 2018), sexually explicit media use (Erickson et al., 2015), drug use patterns (McCarty-Caplan et al., 2014), online platform use (Pravosud et al., 2024), and chemsex (the engagement of sex-drug use) (Scholz-Hehn et al., 2022). This research has identified how behaviors may typologize, suggesting that although it is important to take a variable-centered approach, a person-centered and population-level approach, such as LCA, may a) describe health phenomena for each individual, and b) subset these populations of interest to better tailor and target interventions for subpopulations (Weller et al., 2020). More specifically, one LCA of the data analyzed in the present study identified three profiles of digital platform use (e.g., Multi-venue Users, Non-serosorting App Users, and Serosorting App Users) differing in sexual risk and venue use. This study found that PrEP use and age predicted higher-risk classes, and directed future interventions to tailor HIV prevention to venue-use patterns with targeted messaging across online and physical spaces (Dangerfield et al., 2020).

In this descriptive study, we examine how patterns of social media use, including the specific use of social media platforms (e.g., Twitter, Facebook, Instagram, Snapchat) to seek romantic or sexual partners,on the mental health and sleep health of MSM living in Paris. As Paris has one of the largest lesbian, gay, bisexual, transgender, and queer (LGBTQ+) communities, is one of the safest cities in the world for LGBTQ + people, and has high social media usage, this necessitates further investigation on the relationship between social media, mental health, and sleep hygiene. By investigating patterns of social media engagement and their associations with key psychosocial outcomes, this study aimed to generate novel insights into the potential pathways through which digital technologies shape health among MSM in a European context. Although the data for this study were collected in 2016 using a popular geosocial networking app, the digital landscape has since drastically changed. For example, there has been an emergence of algorithmic-fueled digital applications (e.g., TikTok and Instagram Reels), the rebranding of Twitter into X, and a saturation of social media bots spreading misinformation: this has reshaped how people, including MSM, interact online. Thus, in this study, we describe a social media environment characterized by more stable network-based interactions instead of algorithm-driven short-form videos and content which are more widely used at present. Importantly, this work seeks to expand the scholarly discourse beyond sexual risk behaviors to consider broader dimensions of health equity, recognizing social media as both a resource and a potential risk factor for marginalized populations.

## 2. Methods

### 2.1. Sample recruitment

For recruitment, this study used broadcast advertisements on a popular geosocial-networking smartphone application for MSM, limited to users in the Paris (France) metropolitan area in October 2016. Consistent with previous research (Duncan et al., 2016; Duncan et al., 2018; Goedel and Duncan, 2015), users were shown an advertisement with text encouraging them to click through the advertisement to complete an anonymous web-based survey. To encourage participation, the advertisement described that users who completed the survey were entered in a chance to win €65 (approximately $70 at the time of data collection). The advertisement was placed during three consecutive weekday 24-h periods. The advertisement appeared on the first instance a user logged onto the application in a 24-h period. Precautions (e.g., use of the “Prevent Ballot Box Stuffing” feature on Qualtrics) were taken to avoid and eliminate duplicate responses as done in previous research (Duncan et al., 2016), which resulted in no apparent duplicate responses. The study procedures have been described in detail elsewhere (Duncan et al., 2017b).

The survey included 52 items total. The survey was translated from English into French using an adaptation of the TRAPD (translate, review, adjudicate, pretest, document) translation protocol (Harkness et al., 2003). The survey was translated to French by three native French speakers and then reviewed and adjudicated by an additional native French speaker. The survey was pretested by back-translation by a fifth French speaker who is a health researcher. The survey took an average of 11.4 min (SD = 4.0) for users to complete and was offered in French and English. The vast majority (94.3%) took the survey in French. At the end of the recruitment period, 5206 users had clicked on the advertisement and reached the landing page of the survey, 935 users provided informed consent and began the survey, and 580 users provided informed consent and completed the survey. This represents an overall completion rate of 11.1%. All protocols were approved by the New York University Institutional Review Board prior to data collection.

### 2.2. Social media use measures

Participants were asked whether they had accounts on four major social media platforms: Facebook, Twitter (this social media app was Twitter at the time of data collection, but is now called X), Instagram, and Snapchat (coded as 0 = no, 1 = yes for each platform). Additionally, participants were asked whether they had used any social media platforms in the past six months to find romantic, hookup, or sexual partners (0 = no, 1 = yes), an item adapted from Patel et al. (2016).

### 2.3. Mental health

#### 2.3.1. Depressive symptoms, anxiety symptoms, and psychological distress

The four-item Patient Health Questionnaire (PHQ-4) was used to measure depression, anxiety and other forms of psychological distress (Kroenke et al., 2009) as it has been shown to have good internal consistency and construct validity. The PHQ-4 consists of two subscales: the Patient Health Questionnaire-2 (PHQ-2) for depression and the Generalized Anxiety Disorder Scale (GAD-2) for anxiety. Participants were asked the following question: “Over the last two weeks, how often have you been bothered by the following problems?” The problems for depression were: “Feeling down, depressed, or hopeless” and “Little interest or pleasure in doing things.” The problems for anxiety were: “Feeling nervous, anxious, or on edge” and “Not being able to stop or control worrying.” Response options for all were: “Not at all”; “Several days”; “More than half the days”; and “Nearly every day;” and were rated on a 4-point scale from 0 to 3. The PHQ-2 and GAD-2 scores were dichotomized using cut off of ≥3 for each depression and anxiety symptomology (Kroenke et al., 2009; Kroenke et al., 2010). We chose to dichotomize these scores using the established clinical cutoffs to indicate probable depressive or anxiety symptomology as it has a sensitivity of 83% and a specificity of 92% for detecting major depressive disorder at a cutoff score of ≥3 (Kroenke et al., 2003) and a sensitivity of 86% and a specificity of 83% for detecting generalized anxiety disorder at a cutoff score of ≥3 (Kroenke et al., 2007). Psychological distress was measured using PHQ-4 total score (sample Cronbach's alpha = 0.87) and used the following score ranges to define levels of severity: “None” = 0–2; “Mild” = 3–5; “Moderate” = 6–8; and “Severe” = 9–12. Similarly, the psychological distress score was dichotomized: as either none or mild (score of 0–5) and moderate or severe (score of 6–12) (Kroenke et al., 2009; Kroenke et al., 2010).

### 2.4. Sleep health

#### 2.4.1. Sleep duration, sleep quality and sleep problems

Habitual sleep duration was measured by an item from the widely-used Pittsburgh Sleep Quality Index (PSQI (Buysse et al., 1991; Millar et al., 2019);), which asks, “During the past month, how many hours of actual sleep did you get each night?” Responses were open-ended but were limited to a whole number. We combined responses into short (less than 7 h) versus sufficient (7 h or more; referent) sleep durations, per recommendations from the National Sleep Foundation (Hirshkowitz et al., 2015). Subjective sleep quality was assessed using the final question in the PSQI: “During the past month, how would you rate your sleep quality overall?” (Millar et al., 2019). Response options included: “Very good,” Fairly good,” Fairly bad,” and Very bad.” Given the small number of participants selecting the Very good” and Very bad” options, we collapsed responses into good (referent) versus poor sleep quality. We assessed three sleep problems that are included in the PSQI (Millar et al., 2019): trouble falling asleep, trouble staying awake during wake-time activities, and use of medications to help with sleep. We modified the original PSQI format which asks for frequencies of each problem in a typical week, to a Yes” or No” response format for the following questions: “During the past month, have you experienced any of the following? (1) I had trouble sleeping because I could not get to sleep within 30 min (i.e., problems falling asleep) (2) I had trouble staying awake while driving, eating meals, or engaging in social activity (i.e., problems staying awake during wake-time activities) (3) I took medicine (prescribed or over the counter) to help me sleep.” Due to the low Cronbach's alpha between these three sleep items in this sample (sample Cronbach's alpha = 0.20), we analyzed each problem separately, consistent with previous studies (Duncan et al., 2016; Duncan et al., 2017b; Park et al., 2018).

### 2.5. Stress

Stress was measured with a single item (Park et al., 2018). Participants were presented with a definition of stress as follows: “Stress means a situation in which a person feels tense, restless, nervous or anxious or is unable to sleep at night because his/her mind is troubled all the time.” After this definition, participants responded to the question “Do you feel this kind of stress?” Response options were: “Never” “Rarely,” “Sometimes,” “Often,” and “Always.”

### 2.6. Socio-demographic covariates

We included socio-demographic covariates used in our past studies (Duncan et al., 2016; Duncan et al., 2019). Participants were asked to report their age (in years), which was categorized into five groups: referent = 18–24, 25–29, 30–39, 40–49, and 50 years and older. In addition, participants also reported whether they were born in France (referent = y, no), their sexual orientation (referent = gay, bisexual or another identity), employment status (referent = employed, unemployed, student), and current relationship status (referent = single, relationship with a man).

### 2.7. Statistical analysis

First, we subset our data (92.9%) to only include those participants who had complete responses to the following 14 binary items: sleep quality (sleep duration less than 7 h, bad sleep quality, trouble falling asleep, trouble staying awake during wake-time activities, and use of medications to help with sleep), mental health (i.e., depression as indicated by the PHQ-2, anxiety as indicated by the GAD-2, psychological distress as indicated by the PHQ-4, and stress), and social media use (i.e., use of Facebook, Twitter, Instagram, and Snapchat apps, and use of social media to find romantic, hookup, or sexual partners).

Then, we conducted a three-step LCA to characterize the number of distinct latent classes to best capture patterns of sleep, mental health, and social media usage (Vermunt, 2010). In step one, we identified the number of latent classes which best captured patterns between sleep, mental health, and social media usage. For this step, we first estimated the probability of a participant responding “yes” to each item of each latent class. This allowed us to estimate the posterior probability for each individual belonging to each latent class. In order to identify the latent class model which best fit our data, we conducted a series of five LCAs composed of two to six classes. We assessed each latent model for the following fit indices to identify the best number of classes to fit our data: Bayesian Information Criterion (BIC), Akaike's Bayesian Information Criterion (aBIC), Conditional Akaike Information (cAIC), Vuong-Lo, Mendell, & Rubin Likelihood Ratio Test (VLMR-LRT), and entropy. We assessed class enumeration and model fit based on indices from BIC, aBIC, and cAIC, with lower values indicating a more parsimonious fit (Akaike, 1974; Akaike, 1980; Schwarz, 1978). We then compared each model's fit to the data relative to a model with one less class using the VLMR-LRT, with significant p-values indicating a better fit (Lo et al., 2001; McLachlan and Peel, 2004). Finally, we identified the ability of models to discriminate between classes by comparing entropy values, with entropy values closer to 1 indicating complete certainty of latent class assignment, and values above 0.8 being considered acceptable (Celeux and Soromenho, 1996).

In step two, we assigned participants to their typologized latent class. In step three, we conducted a series of Chi-squared tests to examine associations between sociodemographic and background characteristics and latent class assignment. We used the R environment for analyses (R Core Team, 2025) and the poLCA package to conduct the LCA (Linzer and Lewis, 2011).

## 3. Results

### Description of sample:

Participants belonged to the following age groups: 18–24 years (14.8%), 25–29 years (17.8%), 30–39 years (32.5%), 40–49 years (24.7%), and 50 years or older (10.3%). Most participants identified as gay (86.3%), were born in France (79.4%), were currently employed (68.8%), and reported single relationship status (66.6%). For sleep quality, 30.8% of participants reported poor sleep quality, 28.6% having less than or equal to 6 h of sleep, 45.8% having trouble falling asleep, 12.8% having trouble staying awake during daytime activities, and 5.9% taking medicine to fall asleep. Participants also reported the following mental health indicators: depression (21.9%), anxiety (25.4%), sometimes/often/always feeling stress (69.2%), and moderate or severe psychological distress (22.8%). Of participants, the following reported having accounts on the following social media platforms: Facebook (85.5%), Twitter (38.2%), Instagram (48.2%), and Snapchat (40.4%). Finally, in the last six months, 74.4% of participants used a social media platform to find romantic or sexual partners.

### Results from the Latent Class Analysis:

A total of 580 participants completed the survey. As we received complete LCA item data from 539 participants, this subset of participants was included in the LCA (7.6% missing). We found that the four-class model fit our data best; this model had the lowest BIC and cAIC which suggested that class enumeration (classes = 4) was best for these models. The VLMR-LRT p-value was significant suggesting that the four-class model was a better fit to the data than the three-class model. Additionally, the entropy value was 0.83 suggest more certainty of latent class assignment. Additional information can be found in Table 1.

**Fig. 1.**
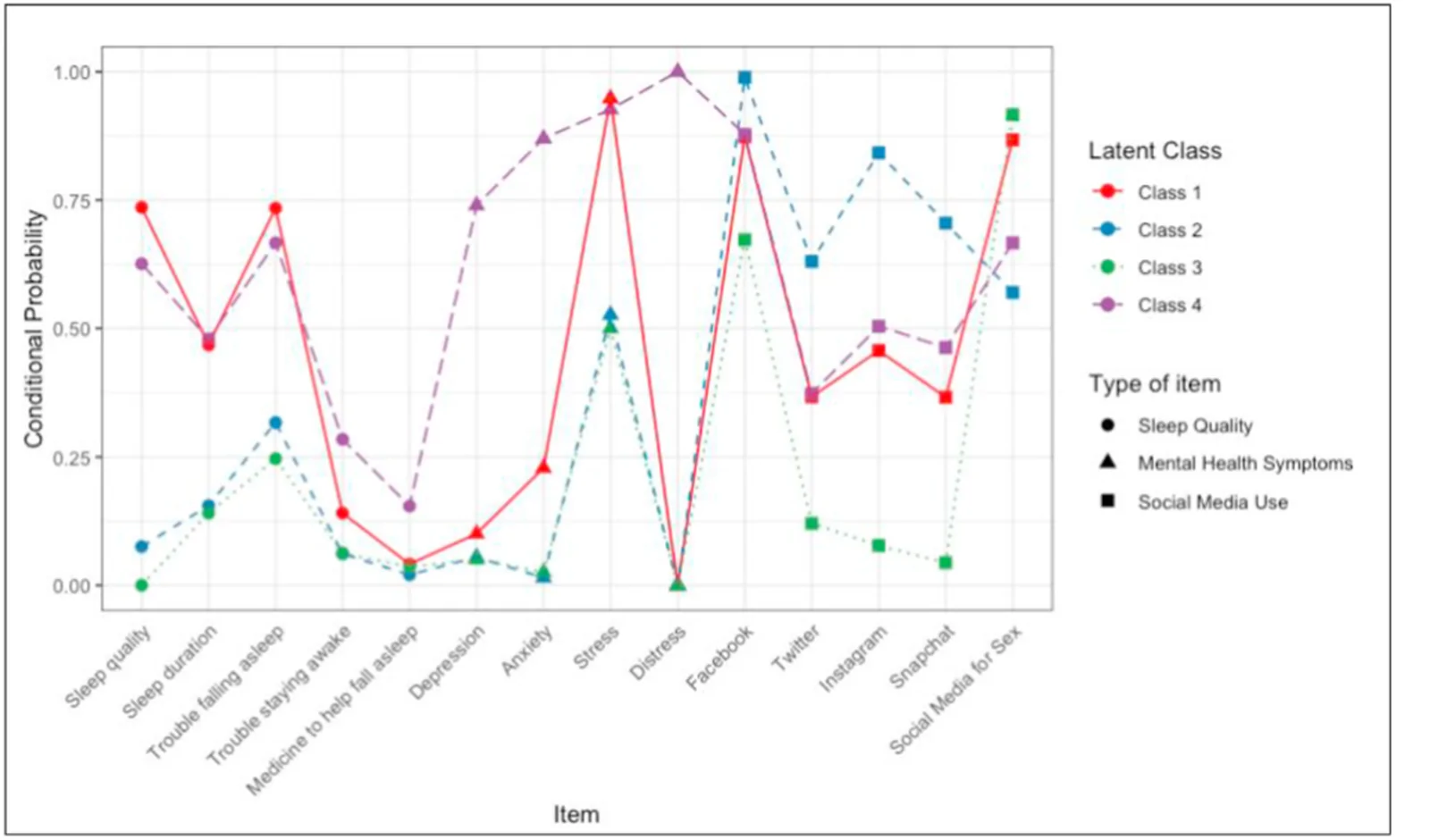
Plot of item conditional probability for the four-class model.

To select the 4-class model, we considered multiple criteria in tandem: fit to data, parsimony, and interpretability (Masyn, 2013; Nylund-Gibson and Choi, 2018). Thus, although aBIC declined through the 6-class solution, BIC and cAIC was lowest and entropy was most stable at the 4-class model. Because one of our criteria included interpretability, we inspected the 5- and 6-class solutions and found that additional classes did not truly indicate new distinct classes but instead seemed to be splits of existing classes. Thus, we selected the 4-class model, and assigned participants into one of four distinct latent classes: 1) *MSM using social media for sex with stress and poor sleep* (n = 97), 2) *MSM using social media generally with good mental and sleep health* (n = 172), 3) *MSM using social media for sex with good mental and sleep health* (n = 147), and 4) *MSM using social media for sex with distress and poor sleep* (n = 123). A plot of the conditional probability of item endorsement, grouped by latent class assignment, can be found in Fig. 1. Additional information about the exact conditional probabilities of item response, by class, can be found in Appendix 1.

To assess evidence of collinearity within classes, we calculated the posterior-weighted within-class correlations. We found that there was low within-class correlation between depression and anxiety (r = − 0.23) and correlations between depression and anxiety, and psychological distress were close to 0 within classes. This suggests that there is no strong residual dependence after conducting the LCA and no substantial collinearity.

### MSM using social media for sex with stress and poor sleep (Class 1):

This group of Facebook-using MSM in Paris had a high conditional probability (>0.75) of using social media for sex and high stress levels. This group also had moderate high conditional probability (>0.60) of poor sleep quality and having trouble falling asleep.

### MSM using social media generally with good mental and sleep health (Class 2):

This group of Facebook-using MSM in Paris had a high conditional probability (>0.75) of using Facebook and Instagram and moderately high conditional probability (≥ 0.60) of using Snapchat and Twitter. This group also had low conditional probability (*<*0.25) of poor or problematic sleep (i.e., poor sleep quality, trouble falling asleep or staying awake, or using medicine to sleep), and mental health symptoms.

### MSM using social media for sex with good mental and sleep health (Class 3):

This group of Facebook-using MSM in Paris had a high conditional probability (>0.75) of using social media for sex, with general social media use as moderately high conditional probability (>0.60) for Facebook and low conditional probability (*<*0.25) for Twitter, Instagram, and Snapchat. This group also had low conditional probability (*<*0.25) of poor or problematic sleep (i.e., poor duration or quality, trouble falling asleep, or using medicine to sleep), and mental health symptoms.

### MSM using social media for sex with distress and poor sleep (Class 4):

This group of Facebook-using MSM in Paris had a moderate high conditional probability (>0.60) of using social media for sex, and high conditional probability (>0.75) of using Facebook. This group also had moderate high conditional probability (>0.60) of poor or problematic sleep (i.e., poor sleep quality, trouble falling asleep). In terms of mental health, this group reported a high conditional probability (>0.75) of psychological distress overall including anxiety symptoms and stress, and a moderate high conditional probability (>0.60) of depressive symptoms.

### Associations between sociodemographic and background characteristics and latent class assignment:

There were statistically significant differences among latent group assignment by age category (p *<* 0.001), employment status (p *<*0.001), all sleep health variables (p *<*0.001), all mental health variables (p *<* 0.001), and all social media usage variables (p *<* 0.001). Additional information regarding associations between sociodemographic and background characteristics and latent class assignment can be found in Table 2.

## 4. Discussion

We found that sleep quality, mental health, and social media use typologized into four distinct latent classes among MSM in Paris, France: MSM using social media for sex with stress and poor sleep, MSM using social media generally with good mental and sleep health, MSM using social media for sex with good mental and sleep health, and MSM using social media for sex with distress and poor sleep. Further, we found that latent group assignment was significantly associated with the sociodemographic characteristics of age and employment.

Generally, we found that most participants used at least one social media platform, with Facebook being the most commonly reported social media platform. Twitter/X was the least commonly used, suggesting that platform selection matters and may be a function of sociodemographic and other contextual factors-future research can consider using more relevant platforms, including those which are less network-focused and more algorithmic and short form video content-focused, for their target populations. We also found that almost 3/4th of MSM in Paris reported using social media to find sexual or romantic partners, suggesting that while geographic proximity may play a role in sexual partner selection, digital sexual networks are important contextual considerations when designing interventions. Considering that MSM who use internet-based venues to meet sexual partners were also more likely to engage in sexual behaviors with higher HIV-related risk (Al-Ajlouni et al., 2018; Goedel and Duncan, 2016), geosocial networking apps may also be considered for future intervention.

We also found that poor sleep quality and trouble falling asleep typologized with using Facebook and using social media to find sexual partners. However, these two groups differed based on their mental health profiles. More specifically, one of the typologies of these groups had a high conditional probability of stress, and the other group had a higher conditional probability of anxiety, stress, psychological distress, and depression. Although Facebook has previously been shown to buffer the effects of acute stress through psychological support (Rus and Tiemensma, 2018), MSM in Paris may be turning to Facebook when they experience trouble falling asleep. Alternatively, Facebook or sexual partner selection on social media activity may increase at night, which could disrupt sleep, resulting in poor sleep quality. Although we did not explore social media use during times of poor sleep, future research may explore how different social media platforms may be used during times of poor sleep or poor mental health. There may also be a biological mechanism explaining the relationship between poor sleep and poor mental health. For example, individuals with poor mental health may experience non-restorative sleep, which can impact sleep quality, and conversely, individuals with poor sleep quality may be at increased risk for poor mental health (Porkka-Heiskanen et al., 2013). Our findings are relevant as poor sleep quality and poor mental health may contribute towards other psychosocial and health risks such as increased substance use and sexual health risks (Sari et al., 2025) in addition to social isolation (Primack et al., 2017) and sedentary time on non workdays (Alley et al., 2016). A future qualitative study could examine the nuances of these typologies to further understand the relationship between sleep, mental health, and social media use.

This finding also contributes to the nascent literature in this area. Zheng et al. (2020) analyzed over 477,000 social media posts from MSM users on the Blued app and found that positive affective states, which peaked in the early morning, were associated with sexual activity, while negative affective states, peaking after midnight, were linked to health concerns, particularly mental health. The findings suggest opportunities to tailor real-time health interventions, such as psychological counseling or sexual health promotion, based on users’ affective states expressed on social media. Gao et al. (2023) found that fear of missing out (FoMO) related to mobile social media significantly predicts depressive symptoms among Chinese college students, with phubbing (i.e., ignoring someone in a social setting by focusing on a smartphone) partially mediating this relationship and social exclusion moderating its direct effect. The results highlight key psychological mechanisms linking FoMO and depression and suggest intervention strategies targeting phubbing and social exclusion to improve student mental health. We are not aware of any studies on the association between social media and sleep health among MSM, with a 2024 systematic review of 184 studies not identifying any studies conducted with MSM populations (Ahmed et al., 2024).

We also identified a group of MSM who had a high conditional probability of using all social media platforms and who did not experience poor mental health or poor sleep: this group was also the largest group across all four classes (n = 172). The second largest group also did not experience poor mental health or poor sleep though used social media for sex (n = 147). As more than half of the sample reporting using social media with mental and sleep wellness, this suggests that social media platforms may also predominantly serve as a space where mental health and well-being is supported, as found in previous studies (Graham et al., 2021; Hiebert and Kortes-Miller, 2021). For example, one study found that Latinx youth in the US turned to diverse social media platforms for mental health support and resources (DuPont-Reyes et al., 2025). Although we did not measure frequency of use, social media in moderation may have utility in supporting mental wellbeing among MSM, which may promote better sleep (Ahmed et al., 2024; Paciente et al., 2024; Rus and Tiemensma, 2018). However, previous studies have found that taking a break from social media may increase wellbeing through improving sleep quality (Graham et al., 2021). One such application which may promote mental health among MSM is TikTok (Paciente et al., 2024), though this app launched in September 2016 and therefore was not yet available during the time of our study. More specifically, during the COVID-19 pandemic, TikTok emerged as a digital home for MSM (Hiebert and Kortes-Miller, 2021). Now, the platform serves as a space for LGBTQ + people to experience community connectedness, which has been previously shown to buffer minority stressors (Paciente et al., 2024). The TikTok algorithmic “For You Page” (FYP) also can affirm LGBTQ + identities; however, as the FYP page may also expose MSM to anti-LGBTQ+ rhetoric (Simpson and Semaan, 2021), it is important to consider weighing the ethics of social media-based interventions. Considering that there are 21.5 million TikTok users over the age of 18 living in France in early 2025, TikTok is the third most popular social media platform after Facebook (31.5 million users) and Instagram (26.6 million users) (Kemp, 2025).

### 4.1. Public health implications

A study with Chinese MSM found that studies which utilized inclusive sampling, such as online surveys, can identify underrepresented latent classes, which may be critical implications for equitable HIV-related interventions (Smith et al., 2019). Our findings contribute to the exant literature and have important implications for public health as European MSM have previously expressed willingness to use social media platforms to better their health. Firstly, our findings emphasize the need for future research with MSM to more accurately measure social media use. For example, our study treats social media application use, sexual partner seeking, mental health, and sleep as dichotomous variables and forfeits more nuanced details regarding intensity, timing, and motivation. Future longitudinal studies or a micro-longitudinal study such as an ecological momentary assessment or passive digital tracking measuring social media use could be developed to further explore causality within these relationships. Second, public health interventions can consider leveraging social media applications that are widely used among MSM such as Facebook, Instagram, Snapchat, and Twitter. When designing interventions, researchers must consider social network theories as well as how subgroups may have different outcomes after receiving an intervention. Social network theories include the theory of homophily, as public health messaging delivered by homophilous individuals to the receiver, especially those on sexual identities, may be uniquely positioned as trusted sources to deliver sensitive information (Byrne, 1971; McPherson et al., 2001; Monge and Contractor, 2003; Schneider et al., 2013; Turner et al., 1987; Shrader et al., 2024a, 2024b). These studies may also be co-designed by MSM in the research process using community-based participatory approaches (Rodriguez et al., 2026).

Interventions for priority populations such as MSM may also consider leveraging the tension between precision science and population health (Khoury et al., 2012). In alignment with Rose's Prevention Paradox, although population level health approaches are most successful in reducing the greatest number of negative health outcomes, they are not as successful as targeted precision health approaches for those populations at greatest risk, and may unintentionally increase health disparities (Rose, 2001). Our findings may contribute to prevention research in that LCA may be one such way to best identify those subgroups at highest risk of negative health outcomes as LCA can go beyond primary prevention and consider the provision of targeted tertiary prevention interventions (Lanza and Rhoades, 2013). Previous interventions have also demonstrated different outcomes based on their LCA subgroup assignment. For example, a selective, personality-targeted intervention to prevent alcohol misuse was found to be effective among youth with high risk personality traits, but only herd effects were seen in low risk personality traits (Conrod et al., 2013). Therefore, a future intervention to improve health among MSM in Paris could consider how different subgroups may be affected, and aim to prioritize those MSM at highest risk of poor sleep and adverse mental health and social media outcomes.

Social media platforms also have the power to engage learners by delivering content to users by allowing them to learn how they want to learn: for this benefit, brick-and-mortar classrooms are turning to social media platforms to teach student learners (Blair and Serafini, 2014). Previous Popular Opinion Leader interventions have found that when a socially influential member of a community communicates HIV risk reduction strategies with their peers, their peers’ behaviors change, but only if they positively perceive the messenger (Group, 2007; Ibrahim et al., 2024; Kelly, 2004; Kelly et al., 1991; Pagkas-Bather et al., 2020; Quinn, 2020). Interventions may be adapted for implementation on social media platforms with Social Media Influencers serving as the Popular Opinion Leader (Alves De Castro and Carthy, 2021), but only if those “messengers” are appropriate and welcomed by the priority population. Also key for these interventions to be successful, Social Media Influencers or community health workers would need to be trained to deliver the chosen evidence-based risk reduction strategies via social media (Ocasio et al., 2026). Also, considering that most social media interventions in a systematic review did not employ a control group, future research can consider more rigorous study designs that employ experimental conditions (Huang et al., 2025).

### 4.2. Study limitations

This study has several important limitations that warrant consideration. First, we used LCA to draw conclusions, and this analytic method is an exploratory approach. We also chose the class enumeration based on model specification selection criteria and chose names for each of the classes- our subjective approach may have introduced bias. There is also a potential of misclassification of latent class assignment. Second, we used a non-probability sampling strategy, which may limit the generalization of findings to the broader population of MSM in France or globally. Additionally, because this study was conducted among MSM in Paris, where there is a large and well-connected LGBTQ + community with relatively high access to digital and mental health resources, our findings may not fully reflect digital engagement patterns among MSM living in more rural regions of France, where social media may serve different functions related to community access and social support. Third, the cross-sectional design of this study limits the ability to draw causal inferences or conclusions as to how these relationships may vary over time as directionality and temporalty between variables cannot be established. To maximize participation, the study included a restricted set of variables, which introduces the potential for residual confounding. For example, more nuanced information about social media use behaviors would be important for future studies to assess including participants' typical time of day of use, reasons for use, length of use, and the type of content that they are exposed to, etc., which are all factors that could plausibly influence both affective states and posting behaviors. Additionally, the study is subject to same-source bias, as both exposure and outcome measures were derived from participants via self-report, increasing the risk of measurement error and shared method variance (van Smeden et al., 2020). Also, not all of our measures were validated among the French population, or MSM in France. Furthermore, we did not include objective measures of behavioral or mental health outcomes, nor did we differentiate explicitly between online and offline behaviors, limiting the specificity of behavioral interpretations. The study also examined a restricted set of social media platforms, potentially limiting generalizability to MSM who engage with other platforms or digital spaces; assessment of the specific language of social media content could be important as well to assess risks and resources across French versus English language social media as a previous study found that content and use varies across languages on social media platforms (DuPont-Reyes et al., 2025). We also acknowledge these data were collected in 2016 and the meaning and function of social media platforms has evolved. For instance, there have been some changes in social media such as the introduction of algorithmic-based applications such as TikTok; therefore, study reproducibility across time and consideration of age/period/cohort effects is important to promoting rigor in this area of research. Considering that our data were collected in 2016, the analyzed sample may not reflect social media users at the time of data collection and presently. Thus instead of interpreting our findings in the context of platform-specific effects, they can be considered as informing social influence mechanisms in digitally mediated social networks. Still, as there are few datasets which capture these measures among MSM with this level of detail, our findings are still empirically relevant and may help foster future innovations in research in this area. Regardless, future studies can consider replicating our study with contemporary samples to promote temporal validity. Finally, selection bias and limited generalizability of study findings should be acknowledged, as MSM participants who engage in social media or specific social media platforms may differ systematically from those who do not, and this relationship may be influenced by age. For example, we found that there were significant differences in latent class assignment between younger MSM and older MSM, which may have affected class membership and interpretation. This may reflect a trend that younger MSM and older MSM may gravitate toward entirely different digital environments.

## 5. Conclusions

Ultimately, this study contributes to a growing body of interdisciplinary research at the intersection of digital technologies, sexual minority health, and urban health. Findings from this work may inform future mediation mechanism studies that enhance causal inference that can in turn help inform culturally tailored interventions, public health messaging, and policy efforts to promote mental and behavioral health among MSM in France and beyond. Future research may also consider exploring these relationships in other settings, including with different social media platforms. It is also important that these efforts identify opportunities to leverage social media as a platform for health promotion in digitally connected communities as social media use was overwhelmingly used safely in the current study.

## Supplementary Material

1

## Figures and Tables

**Table 1:** Model fit indices for 2–6 classes.

Model	Log-likelihood	Res. DoF^a^	BIC^b^	aBIC^c^	cAIC^d^	Likelihood-ratio	VLMR-LRT^e^	VLMR-LRT p-value	Entropy

2 class model	−3801	510	7783.47	7691.42	7812.47	1785.44	741.024	<0.001	0.89
3 class model	−3669	495	7614.98	7475.30	7658.98	1522.60	249.615	<0.001	0.82
4 class model	−3605	480	7581.92	7394.64	7640.92	1395.20	120.984	<0.001	0.83
5 class model	−3575	465	7616.08	7381.17	7690.08	1335.00	57.166	<0.001	0.82
6 class model	−3552	450	7663.29	7380.77	7752.29	1287.87	44.762	<0.001	0.79

a Residual Degrees of Freedom.

b Bayesian Information Criterion.

c Sample-Size Adjusted Bayesian Information Criterion.

d Conditional Akaike Information Criteria.

e Vuong-Lo, Mendell, & Rubin Likelihood Ratio Test.

**Table 2:** Associations between sociodemographic and background characteristics and latent class assignment.

	MSM using social media for sex with stress and poor sleep	MSM using social media generally with good mental and sleep health	MSM using social media for sex with good mental and sleep health	MSM using social media for sex with distress and poor sleep	P-value
					
	(N = 97)	(N = 172)	(N = 147)	(N = 123)	

**Age**					
18–24 years	13 (13.4%)	42 (24.4%)	5 (3.4%)	20 (16.3%)	<0.001
25–29 years	16 (16.5%)	35 (20.3%)	14 (9.5%)	31 (25.2%)	
30–39 years	34 (35.1%)	55 (32.0%)	47 (32.0%)	39 (31.7%)	
40–49 years	24 (24.7%)	31 (18.0%)	53 (36.1%)	25 (20.3%)	
50 years or older	10 (10.3%)	9 (5.2%)	26 (17.7%)	8 (6.5%)	
Missing	0 (0.0%)	0 (0.0%)	2 (1.4%)	0 (0.0%)	
**Sexual orientation**					
Gay	88 (90.7%)	147 (85.5%)	125 (85.0%)	105 (85.4%)	0.602
Bisexual or another identity	9 (9.3%)	25 (14.5%)	21 (14.3%)	18 (14.6%)	
Missing	0 (0.0%)	0 (0%)	1 (0.7%)	0 (0.0%)	
**France nativity**					
Born in France	75 (77.3%)	136 (79.1%)	120 (81.6%)	97 (78.9%)	0.805
Not born in France	22 (22.7%)	36 (20.9%)	26 (17.7%)	26 (21.1%)	
Missing	0 (0.0%)	0 (0.0%)	1 (0.7%)	0 (0%)	
**Employment status**					
Employed	76 (78.4%)	113 (65.7%)	108 (73.5%)	74 (60.2%)	0.001
Student	13 (13.4%)	35 (20.3%)	8 (5.4%)	22 (17.9%)	
Unemployed	8 (8.2%)	24 (14.0%)	26 (17.7%)	24 (19.5%)	
Missing	0 (0.0%)	0 (0.0%)	5 (3.4%)	3 (2.4%)	
**Relationship status**					
Single	64 (66.0%)	122 (70.9%)	89 (60.5%)	84 (68.3%)	0.287
Relationship with a man	33 (34.0%)	46 (26.7%)	53 (36.1%)	35 (28.5%)	
Relationship with a woman	0 (0.0%)	3 (1.7%)	4 (2.7%)	4 (3.3%)	
Missing	0 (0.0%)	1 (0.6%)	1 (0.7%)	0 (0.0%)	
**Sleep health**					
Poor sleep quality	79 (81.4%)	10 (5.8%)	0 (0.0%)	77 (62.6%)	<0.001
Poor sleep duration	48 (49.5%)	26 (15.1%)	21 (14.3%)	59 (48.0%)	<0.001
Trouble falling asleep	74 (76.3%)	53 (30.8%)	38 (25.9%)	82 (66.7%)	<0.001
Trouble staying awake during daytime activities	14 (14.4%)	10 (5.8%)	10 (6.8%)	35 (28.5%)	<0.001
Took medicine to fall asleep	4 (4.1%)	3 (1.7%)	6 (4.1%)	19 (15.4%)	<0.001
**Mental health**					
Depression	10 (10.3%)	9 (5.2%)	8 (5.4%)	91 (74.0%)	<0.001
Anxiety	25 (25.8%)	1 (0.6%)	4 (2.7%)	107 (87.0%)	<0.001
Stress	94 (96.9%)	89 (51.7%)	76 (51.7%)	114 (92.7%)	<0.001
Psychological distress	0 (0.0%)	0 (0.0%)	0 (0.0%)	123 (100.0%)	
**Social media usage**					
Facebook	85 (87.6%)	170 (98.8%)	98 (66.7%)	108 (87.8%)	<0.001
Twitter	37 (38.1%)	103 (59.9%)	20 (13.6%)	46 (37.4%)	<0.001
Instagram	52 (53.6%)	21 (12.2%)	145 (98.6%)	61 (49.6%)	<0.001
Snapchat	37 (38.1%)	119 (69.2%)	5 (3.4%)	57 (46.3%)	<0.001
Uses social media to find sexual partners	84 (86.6%)	100 (58.1%)	135 (91.8%)	82 (66.7%)	<0.001

## Appendix A. Supplementary data

Supplementary data to this article can be found online at https://doi.org/10.1016/j.ssmmh.2026.100615.
