# Supplementary material for: Hookups, hashtags, and health: A Latent Class Analysis of social media, mental health and sleep among MSM in Paris, France
Source: SSM Ment Health. Author manuscript; Available in PMC 2026 Aug 26. (PMC13505967; doi:10.1016/j.ssmmh.2026.100615)
Supplement: Supplementary file 1 [file NIHMS2187954-supplement-1.docx]

**Appendix 1:** Conditional probability of item response, by class

| **Item** | **Stressed Facebook users and social media sex seekers with poor sleep** | **Well slept and mentally well MSM who use social media** | **Well slept and mentally well MSM who use Facebook for sex** | **Psychologically distressed and Facebook-using MSM** |
| --- | --- | --- | --- | --- |
| **Poor sleep quality** | 0.74 | 0.08 | 0.00 | 0.63 |
| **Poor sleep duration** | 0.47 | 0.16 | 0.14 | 0.48 |
| **Trouble falling asleep** | 0.73 | 0.32 | 0.25 | 0.67 |
| **Trouble staying awake** | 0.14 | 0.06 | 0.06 | 0.28 |
| **Medicine to help fall asleep** | 0.04 | 0.02 | 0.04 | 0.15 |
| **Depression** | 0.10 | 0.05 | 0.05 | 0.74 |
| **Anxiety** | 0.23 | 0.02 | 0.03 | 0.87 |
| **Stress** | 0.95 | 0.53 | 0.50 | 0.93 |
| **Distress** | 0.00 | 0.00 | 0.00 | 1.00 |
| **Facebook** | 0.87 | 0.99 | 0.67 | 0.88 |
| **Twitter** | 0.37 | 0.63 | 0.12 | 0.37 |
| **Instagram** | 0.46 | 0.84 | 0.08 | 0.50 |
| **Snapchat** | 0.37 | 0.71 | 0.04 | 0.46 |
| **Social media for sex** | 0.87 | 0.57 | 0.92 | 0.67 |
